# Limb Salvage With Single-Stage Bilateral Pantalar Arthrodesis for Severe Equinovarus in Parkinson’s Disease: A Case Report

**DOI:** 10.7759/cureus.94566

**Published:** 2025-10-14

**Authors:** Jannani Krishnan, James B Warne

**Affiliations:** 1 Podiatric Medicine and Surgery, VA Palo Alto Health Care Systems, Palo Alto, USA

**Keywords:** ankle deformity correction, bilateral ankle fusion, foot and ankle reconstruction, intramedullary nail fixation, lower extremity limb salvage, pantalar arthrodesis, retrograde nailing, rigid equinovarus deformity, tibiotalocalcaneal arthrodesis

## Abstract

Fixed bilateral equinovarus deformities are uncommon and difficult to manage, particularly in patients with neurodegenerative disorders affecting lower extremity neuromuscular function and gait, such as Parkinson’s disease. The purpose of this case report is to present the surgical correction and functional outcomes of a patient with severe, fixed bilateral equinovarus deformities treated with single-stage bilateral pantalar arthrodesis using intramedullary (IM) nail fixation. A 75-year-old man with a history of Parkinson’s disease and lumbar spine compression fractures presented with progressing bilateral equinovarus deformity resulting in loss of ambulation. After failing conservative treatment, the patient underwent a single-staged bilateral pantalar arthrodesis. Surgical intervention involved soft tissue releases, osteotomies, and arthrodesis using an IM nail. Postoperative care included non-weight-bearing, pain management, wound care, physical therapy, and custom bracing. Patient outcomes were assessed using the Visual Analog Scale (VAS), American Orthopaedic Foot and Ankle Society (AOFAS) Hindfoot scores, functional capacity, and gait analysis. The patient progressed from non-ambulatory status to walking with double-upright metal AFO braces. VAS pain score improved from 6/10 preoperatively to 3/10 postoperatively. AOFAS Hindfoot scores increased from 0/100 preoperatively to 49/100 nine months postoperatively, with improvement across pain, function, and alignment domains. Single-stage bilateral pantalar arthrodesis with IM nail fixation is a viable surgical option for patients with severe, fixed equinovarus deformities, especially when conservative measures fail. This technique provides durable correction, pain relief, and functional improvement, despite the inherent risk associated with extensive soft tissue releases and osseous corrections in a bilateral case. Careful patient selection and close postoperative monitoring are essential to achieving successful outcomes.

## Introduction

Fixed equinovarus deformity is a complex condition that can result from neuromuscular and musculoskeletal pathologies. In Parkinson’s disease, the rigidity, dystonia, and abnormal neuromuscular control can contribute to bilateral plantarflexion ankle deformities with clawing of the toes. Consequently, this deformity can hinder ambulation and cause pressure ulcers and pain [1]. Patients may struggle with walking, standing for prolonged periods, or fitting in shoes, which can significantly impact independence and quality of life (QOL). Although rare, compression fractures can impair motor function by affecting specific nerve roots, causing weakness and loss of sensation to the lower extremities [2].

A thorough understanding of the pathomechanics of bilateral foot and ankle deformity is essential for appropriate management. Detailed history and physical examination help assess severity and functional limitations. Initial treatment involves conservative measures such as orthotics, bracing, physical therapy, injections, or serial casting, which may offer temporary relief. Recent literature highlights the interplay between proprioceptive feedback and postural control in Parkinson’s disease. Another rehabilitation strategy called automated mechanical peripheral stimulation (AMPS) has shown promising results in improved gait velocity, stride length, and stability by enhancing neuromuscular coordination [3]. In rigid or severe deformities, surgical correction, such as tendon lengthening, tenotomies/tendon transfers, osteotomies, or arthrodesis, is often required to achieve a functional, plantigrade, and braceable foot. The goals are to reduce pain, restore alignment, and prevent risk of skin breakdown [4].

Bilateral pantalar arthrodesis is a challenging procedure indicated for severe and hindfoot deformities, often arising from trauma, osteoarthritis, or neurological disorders [5]. It involves the fusion of the tibiotalocalcaneal, talonavicular, and calcanealcuboid joints to restore alignment, stabilize the foot and ankle, and alleviate pain. While unilateral pantalar arthrodesis is well-established, bilateral cases are rare and require meticulous planning due to the impact on mobility, rehabilitation, and surgical risk. Indications for pantalar arthrodesis include severe pain, deformity, and instability. Contraindications include sepsis or active infection, severe vascular disease, or notable malalignment of the tibia as a result of trauma [6]. Comprehensive preoperative planning and counseling of the patient and family regarding postoperative recovery time, gait impairment, and rehabilitation challenges are crucial.

Intramedullary (IM) nail fixation offers a distinct advantage for the targeted tibiotalocalcaneal (TTC) component of pantalar arthrodesis, including its superior biomechanical stability, minimal soft tissue dissection, and favorable alignment and healing. Since the IM nail is inserted within the medullary bone of the tibia, talus, and calcaneus, surgeons can achieve robust correction with minimal disruption to surrounding soft tissue and neurovascular structures. Sustained compression can be achieved through the tibiotalar and subtalar joints after surgery when utilizing IM nail fixation [7]. Traditional plating may not achieve adequate stability or correction, as some deformities are more severe than others, in which IM nail fixation is considered. Several studies have described the biomechanical impact and surgical techniques for TTC fusion, including the use of internal fixation such as pins, plates and screw, IM nail, and bone grafting and external fixation [8,9]. In the present case, IM nail fixation was used to stabilize the TTC component, while additional fusion of the talonavicular and calcaneocuboid joints was performed with staple fixation, thereby achieving pantalar arthrodesis.

To our knowledge, no prior studies have documented single-stage bilateral pantalar arthrodesis using an IM nail approach for a bilateral fixed cavovarus deformity. This case highlights the importance of individualized surgical planning, considering patient-specific goals, comorbidities, and social support, and contributes to the limited literature on IM nail fixation for bilateral pantalar arthrodesis.

## Case presentation

Case description

A 75-year-old man with a past medical history of Parkinson’s disease, ventricular tachycardia, hypertension, hearing loss, and chronic lower back pain presented with bilateral fixed equinovarus deformity. Approximately two years prior to presentation, the patient sustained a fall, resulting in lumbar compression fractures of the L3 and L4 vertebrae. Several months later, he experienced worsening gait instability, characterized by dragging of the feet and increasing difficulty in ambulation. His feet progressively began to turn inward, ultimately leading to the complete loss of independent mobility.

The patient was evaluated by other specialties but was lost to follow-up and underwent a trial of physical therapy, which yielded no improvement. Due to the severity of the bilateral deformities and progressive functional decline, bracing alone was deemed insufficient, and below-knee amputation was initially recommended. However, the patient and caregivers expressed a strong desire to pursue limb salvage to facilitate transfers and regain mobility. He was then referred to the podiatric surgery department for further evaluation and surgical planning.

**Figure 1:**
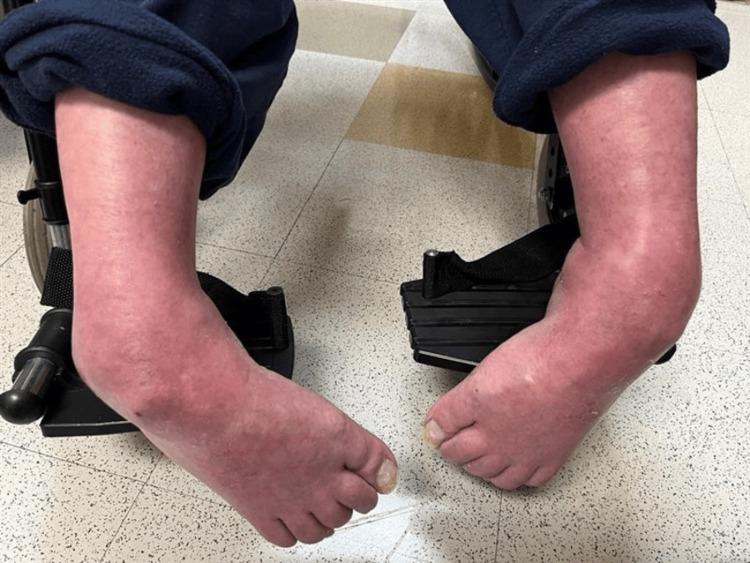
Preoperative clinical photograph Clinical photograph demonstrating severe and rigid bilateral equinovarus deformities. The patient presents with plantarflexed and inverted foot positioning, consistent with fixed contracture and chronic neuromuscular imbalance, leading to significant functional impairment and loss of ambulatory capacity.

Physical examination revealed bilateral equinovarus deformity with both feet in a rigid plantarflexed position. Additionally, there was notable adductovarus contracture from the forefoot, contributing to the inward-facing orientation of the feet. The talar head was prominently palpable along the lateral aspect of both feet. The ankle joint range of motion was severely restricted. The toes demonstrated flexion contractures consistent with the long-standing nature of the deformities. Muscle tone and rigidity were increased; however, deep tendon reflexes were preserved, and no focal sensory deficits were noted. The skin overlaying deformities were intact, with no evidence of ulceration, breakdown, or ischemia, although mild erythema and swelling were observed, likely secondary to chronic irritation from shoe gear and from prolonged dependent position (Figure 1).

**Figure 2:**
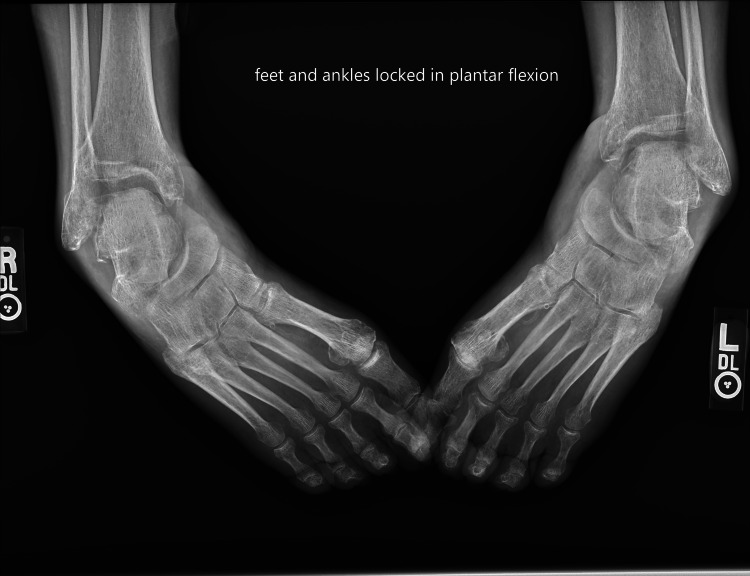
Preoperative AP radiograph of the bilateral ankle and hindfoot Preoperative radiograph demonstrating bilateral hindfoot deformities with overall rigid plantarflexion and adductovarus deformity. AP: anteroposterior

As shown in Figure 2, preoperative radiographs confirmed bilateral adductovarus deformity of the hindfoot with preserved tibial alignment, talar lateral deviation, talonavicular uncovering, and midfoot and forefoot adduction.

**Figure 3:**
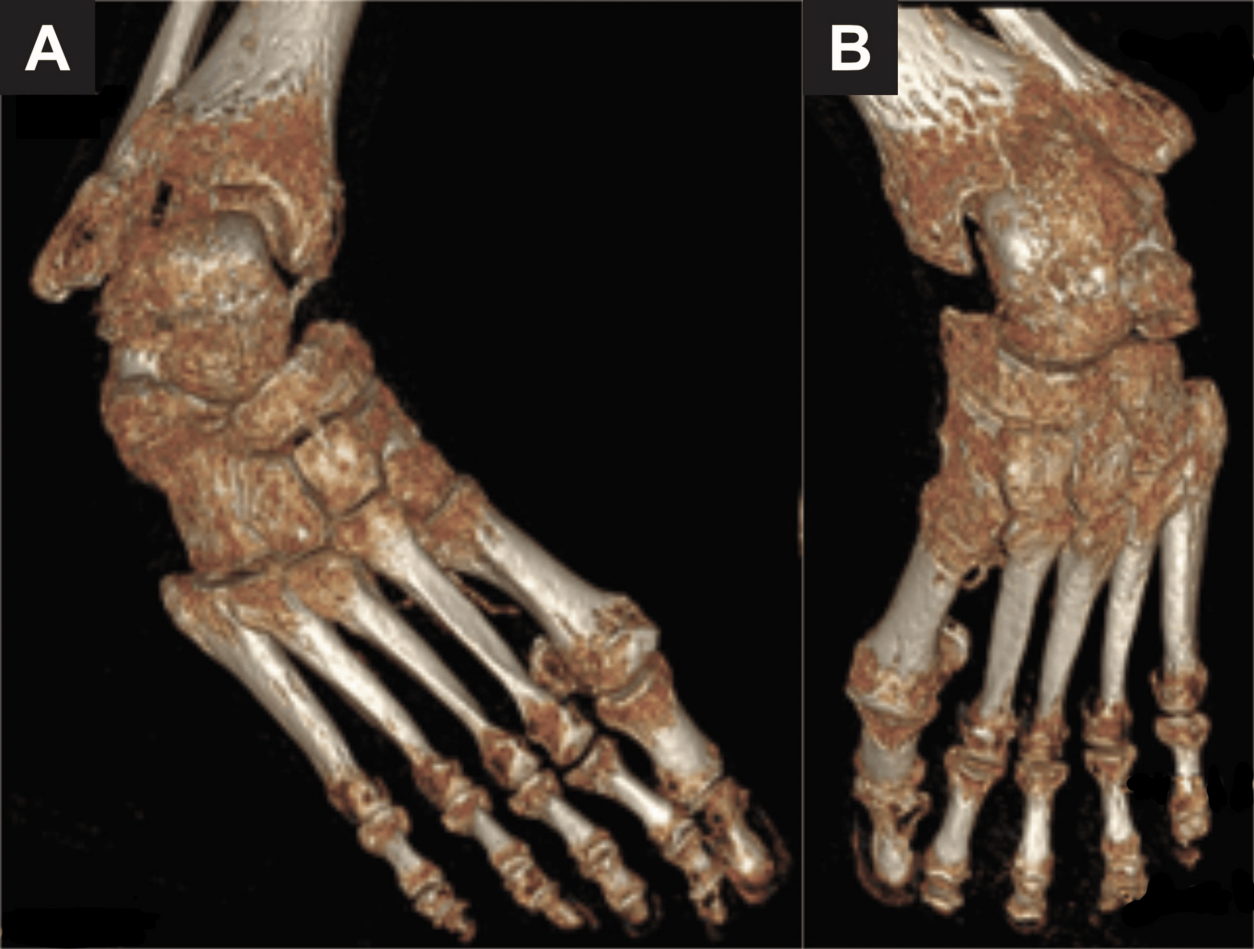
Preoperative CT of bilateral feet A: Right foot, B: left foot Three-dimensional reconstructed CT images demonstrating the right (A) and left (B) feet showing severe, rigid equinovarus deformities. Both sides reveal marked joint incongruity, midfoot collapse, and significant degenerative changes at the tibiotalar and subtalar joints. CT: computed tomography

Computed tomography (CT) imaging supported the severity of deformity and demonstrated significant hindfoot adduction and peritalar subluxation. There is marked collapse of the tibiotalar and subtalar articulation with evidence of talonavicular and calcaneocuboid joint degeneration (Figure 3A-3B).

Surgical technique

The patient was placed in a supine position with the bilateral lower extremities prepped and draped in a sterile fashion. General anesthesia was administered, and a thigh tourniquet was applied and inflated to 300 mmHg to one extremity at a time.

Left Lower Extremity

**Figure 4:**
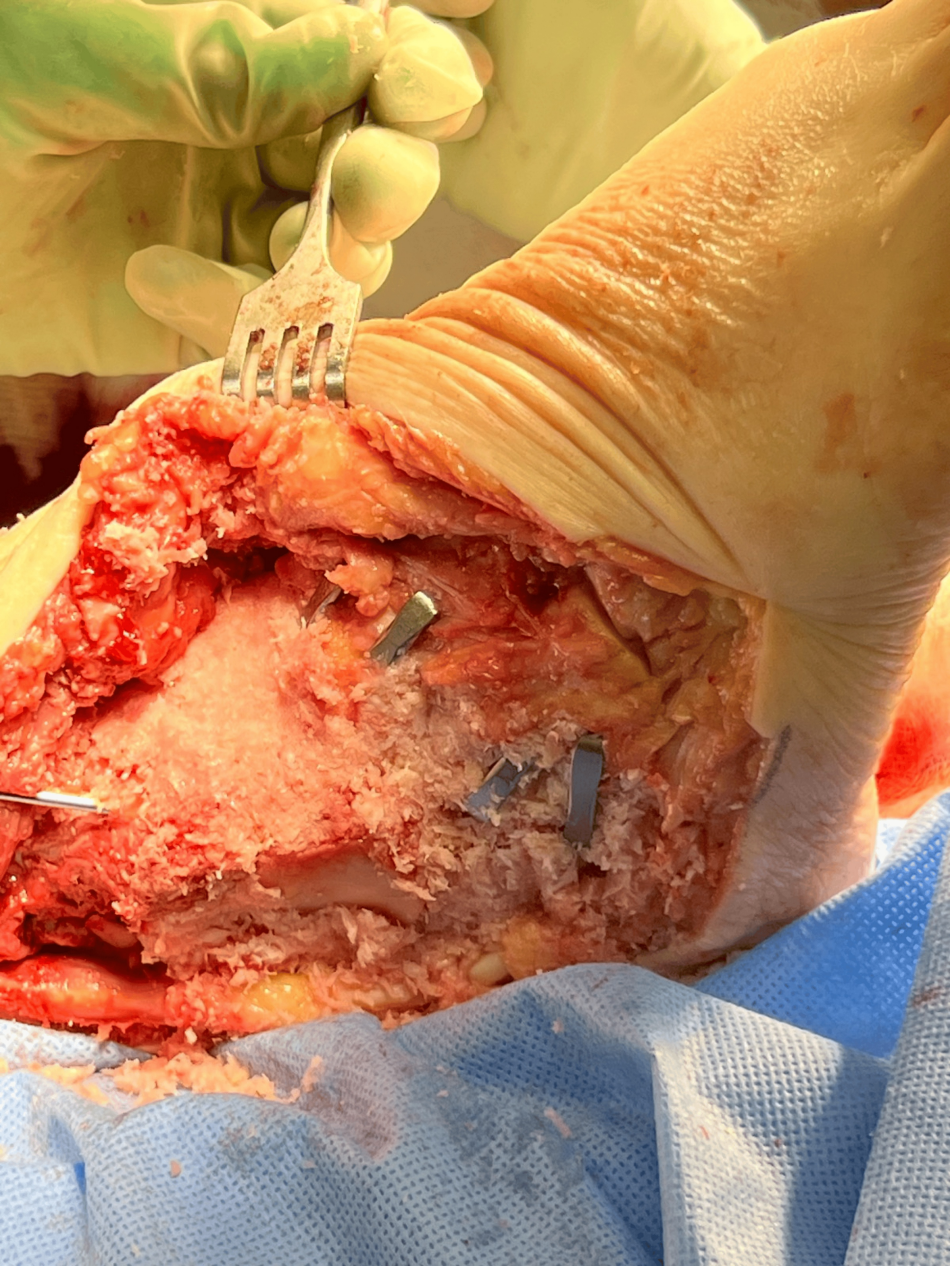
Intraoperative image of triple arthrodesis with stable fixation Intraoperative view demonstrating fusion of STJ, TNJ, and CCJ using staple fixation. Joints were prepared with cartilage resection, subchondral bone decortication, and cancellous bone chips prior to compression and fixation. STJ: subtalar joint, TNJ: talonavicular joint, CCJ: calcaneocuboid joint

Bone marrow aspirate was obtained from the tibial tuberosity and set aside for the joint preparation stage. Soft tissue procedures were performed first to address contractures. This began with an Achilles tenotomy, followed by a posterior medial release involving the release of the posterior tibial tendon and medial band of the plantar fascia. Due to the increased skin gaping from severe tissue contracture, Neox RT (Amniox Medical, Inc., Miami, FL), a cryopreserved umbilical cord allograft with regenerative and anti-inflammatory properties, was applied to the defect and secured with sutures. Despite the soft tissue corrections, residual deformity was noted. To address the osseous component, a curvilinear lateral incision was created, and a transfibular takedown was performed to improve visualization of the ankle and subtalar joints. This exposure also facilitated nail passage and preparation of the tibiotalar joint surfaces for arthrodesis. Access to the talonavicular and calcaneocuboid joints was achieved through extension of the lateral incision distally into the sinus tarsi and lateral column, which provided adequate visualization of the midtarsal complex. A combination of a drill bit and osteotome was utilized to denude the articular cartilage and prepare the subchondral surfaces of the joints. Correction was achieved by performing wedge osteotomies at the talonavicular and calcaneocuboid joints, targeting the midtarsal joint complex. The joints were packed with a mixture of bone marrow aspirate and ArthroCell (Arthrex Inc., Naples, FL), a cryopreserved allogenic bone graft containing viable cells intended to support bone healing. To achieve proper joint compression, Arthrex DynaNite Staples (Arthrex Inc., Naples, FL) were inserted into the talonavicular and calcaneocuboid joints (Figure 4).

The entry point for the intramedullary nail was determined using intraoperative fluoroscopy, guided by lateral and calcaneal axial views to align the tibial long axis and calcaneal bisecting line, ensuring proper trajectory without the need for direct measurements [10].

Temporary fixation was achieved using Kirschner wires placed in the desired alignment and correction, with proper positioning confirmed under fluoroscopic guidance. TTC arthrodesis was performed using the Dual Compression Hindfoot Nail (Arthrex Inc., Naples, FL). The tourniquet was released prior to the sequential reaming process to reduce the risk of thermal necrosis. A 10.5 mm × 180 mm nail was inserted in accordance with the Arthrex Dual Compression Hindfoot Nail surgical technique guide (Arthrex Inc., Naples, FL). Compression screws were inserted as follows: two in the calcaneus, one in the talus, and two in the proximal tibia to achieve proper alignment and a sturdy construct. Intraoperative fluoroscopy was used to confirm the final position and alignment.

Right Lower Extremity

A thigh tourniquet was applied and inflated to 300 mmHg to the right lower extremity. Bone marrow aspirate was obtained from the tibial tuberosity and set aside for the joint preparation stage. Achilles tenotomy was performed, followed by a Z-plasty of the skin to facilitate adequate skin closure due to severe contracture. The remaining surgical steps were performed identically to the left lower extremity, including joint preparation, bone grafting, joint fixation, and insertion of an intramedullary nail. Intraoperative fluoroscopy confirmed the alignment and stability of the construct. Lastly, a layered closure was carried out, and bilateral walking boots were properly fitted.

**Figure 5:**
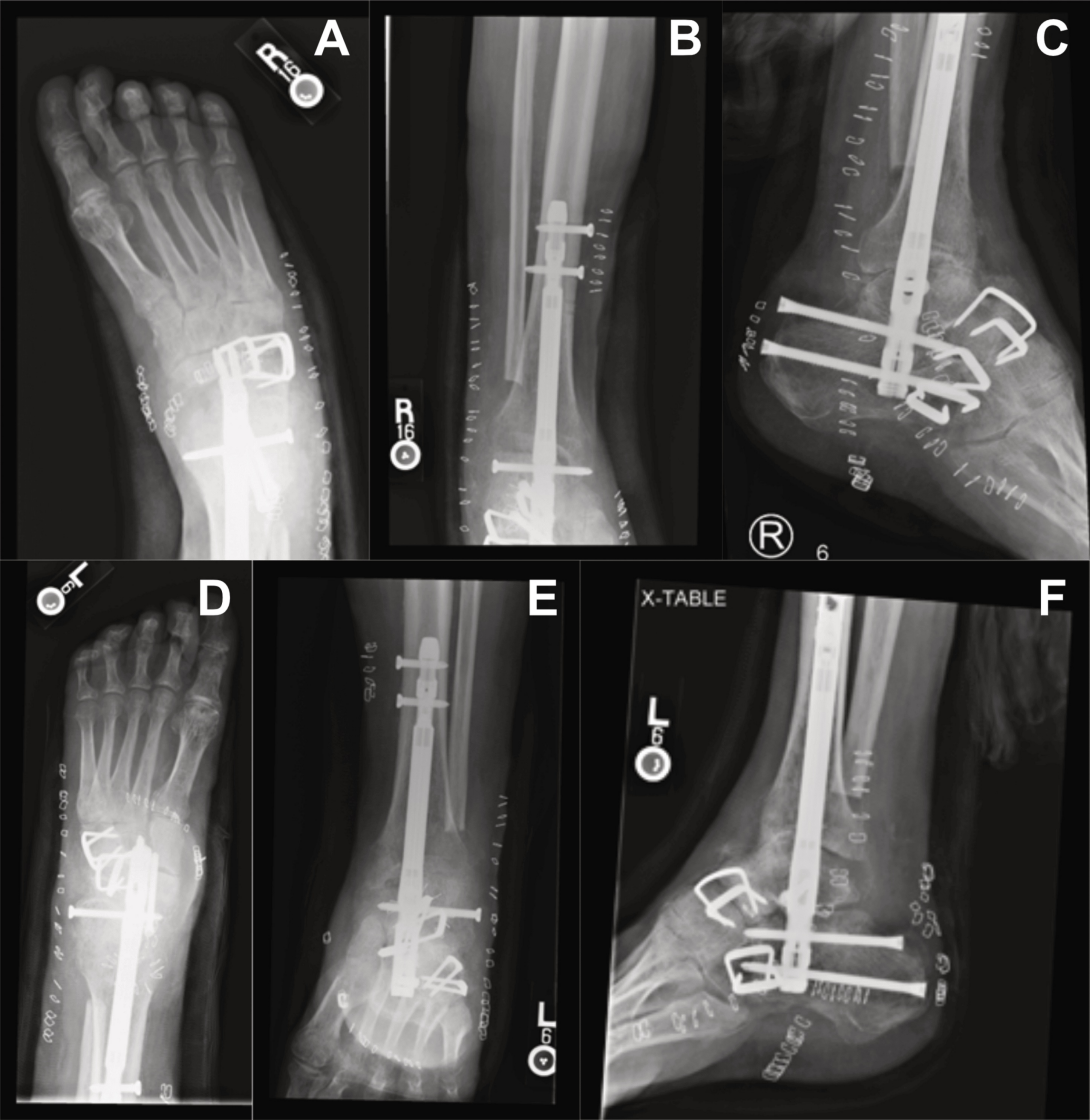
Postoperative radiographic views following bilateral pantalar arthrodesis A: Right foot anteroposterior view, B: right ankle anteroposterior view, C: right ankle lateral view, D: left foot anteroposterior view, E: left ankle anteroposterior view, F: left ankle lateral view

**Figure 6:**
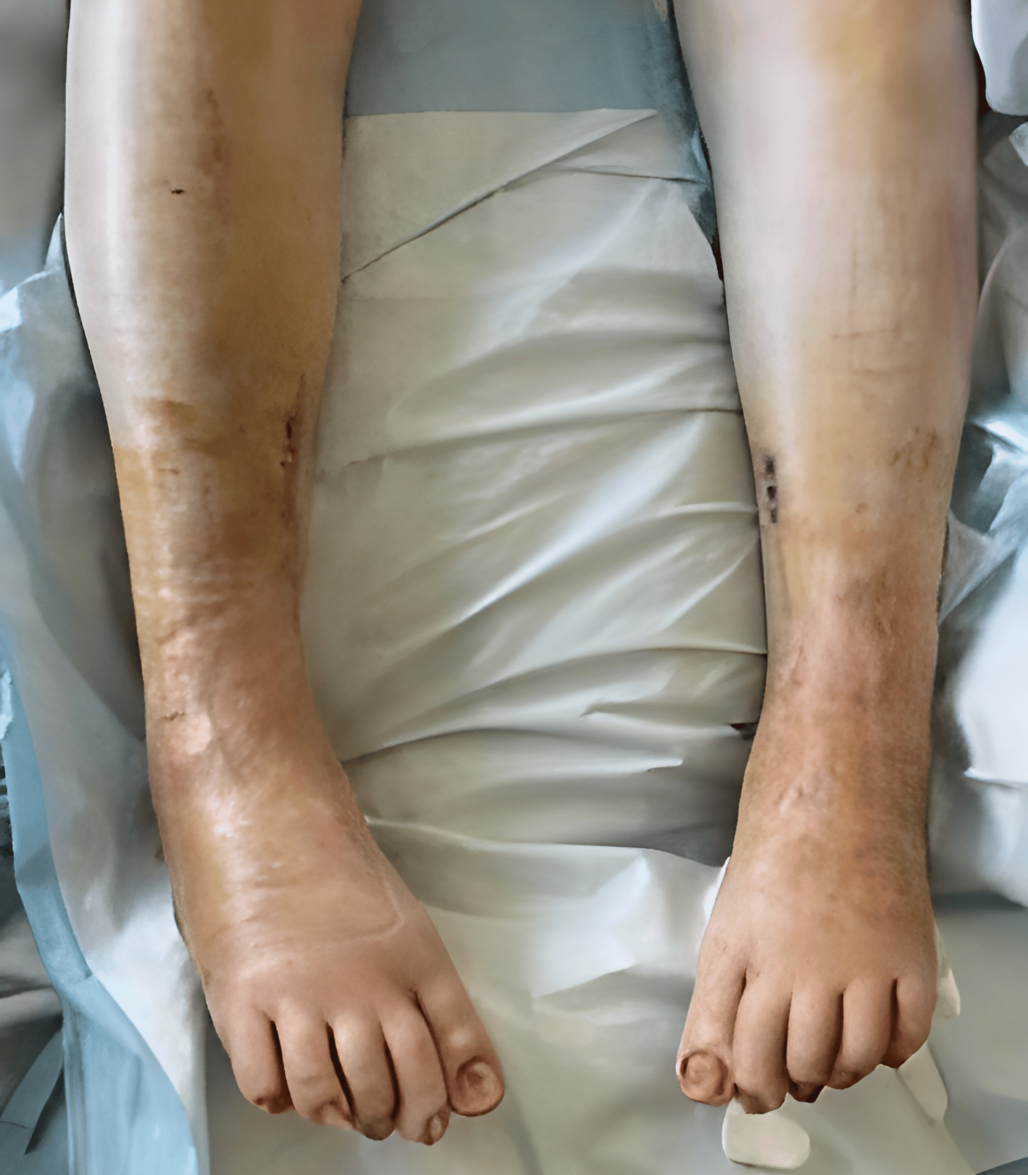
Postoperative clinical image of bilateral pantalar arthrodesis

Postoperative films (Figure 5A-5F) and clinical pictures (Figure 6) were obtained to evaluate the placement of hardware, alignment, and overall deformity correction.

Intramedullary nail implant details

**Figure 7:**
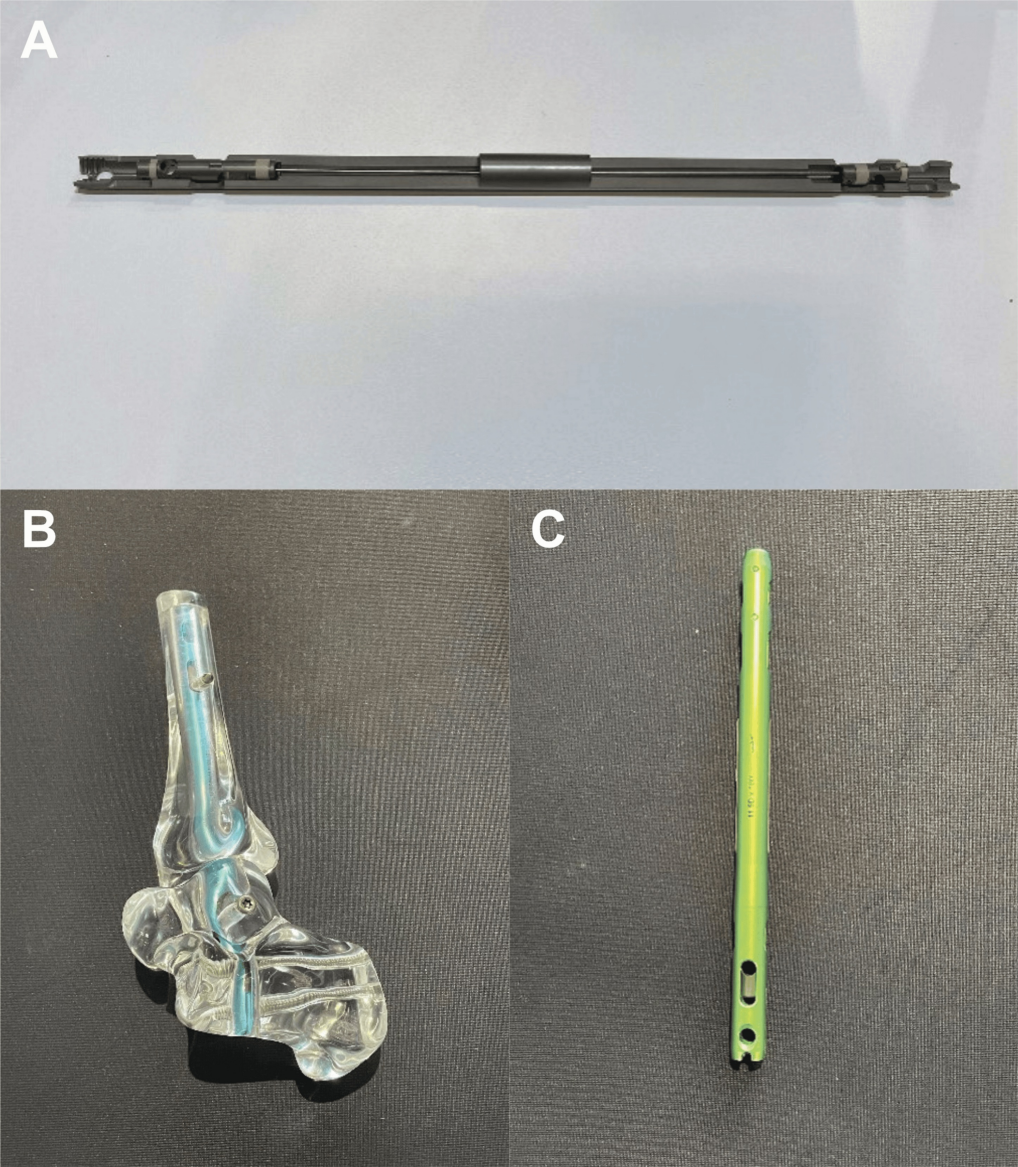
Dual hindfoot nail system components A: Bisection of the dual hindfoot nail demonstrating the internal compression mechanism and cannulated design, B: implant model showing the overall fit into the foot and ankle, C: standalone view of the assembled implant showing the design for stable fixation across the tibia, talus, and calcaneus

The Arthrex Dual Compression Hindfoot Nail is designed to provide both intraoperative and sustained postoperative compression, which is critical for achieving successful fusion in tibiotalocalcaneal arthrodesis. The construct consists of a retrograde intramedullary nail inserted through the calcaneus and advanced proximally into the talus and tibia, with an interlocking screw placed across the calcaneus, talus, and tibia to minimize micromotion at the fusion sites. After the alignment is achieved, the inner nitinol core is stretched under load, creating dynamic, sustained compression across the tibiotalar and subtalar joints, unlike traditional IM nail systems, which rely on static, screw-based compression that may decrease over time due to bone resorption. This design is particularly advantageous in cases involving poor bone quality or complex hindfoot deformity, as in the current case, where a durable alignment and long-term stability were essential (Figure 7A-7C).

Methods

The patient was evaluated postoperatively in terms of recovery and outcome. Pain and patient satisfaction were measured preoperatively and at one, six, and nine months postoperatively using the Visual Analog Scale (VAS), an open-access tool. Clinical outcomes were assessed using the American Orthopaedic Foot and Ankle Society (AOFAS) Ankle-Hindfoot Scale, a validated clinician-reported scoring system that evaluates pain (40 points), function (50 points), and alignment (10 points), for a total score of 100 (Table 1). AOFAS scores were again collected at the same points: preoperatively and at one, six, and nine months postoperatively. The AOFAS scoring system was used with permission and is reproduced from the original publication by Kitaoka et al. [11].

**Table 1:** AOFAS Ankle-Hindfoot scoring system Adapted from Kitaoka et al. [11] with permission from SAGE Publications

Pain (40 points)	
None	40
Mild, occasional	30
Moderate, daily	20
Severe, almost always present	0
Function (50 points)	
Activity limitation, support requirement	
No limitation, no support	10
No limitation of daily activities, limitation of recreational activities, no support	7
Limited daily and recreational activities, cane	4
Severe limitation of daily and recreational activities, walker, crutches, wheelchair, brace	0
Maximum walking distance, blocks	
Greater than 6	5
4-6	4
1-3	2
Less than 1	0
Walking surfaces	
No difficulty on any surface	5
Some difficulty on uneven terrain, stairs, inclines, ladders	3
Severe difficulty on uneven terrain, stairs, inclines, ladders	0
Gait abnormalities	
None, slight	8
Obvious	4
Marked	0
Sagittal motion (flexion plus extension)	
Normal or mild restriction (30° or more)	8
Moderate restriction (15°-29°)	4
Severe restriction (less than 29°)	0
Hindfoot motion (inversion plus eversion)	
Normal or mild restriction (75%-100% normal)	6
Moderate restriction (25%-74% normal)	3
Marked restriction (less than 25% normal)	0
Ankle-hindfoot stability (anteroposterior, varus-valgus)	
Stable	8
Deformity unstable	0
Alignment (10 points)	
Good, plantigrade foot, ankle-hindfoot well aligned	10
Fair, plantigrade foot, some degree of ankle-hindfoot malalignment observed, no symptoms	5
Poor, nonplantigrade foot, severe malalignment, symptoms	0

Postoperative course and outcomes

Postoperative Rehabilitation and Orthotic Bracing Strategies

**Figure 8:**
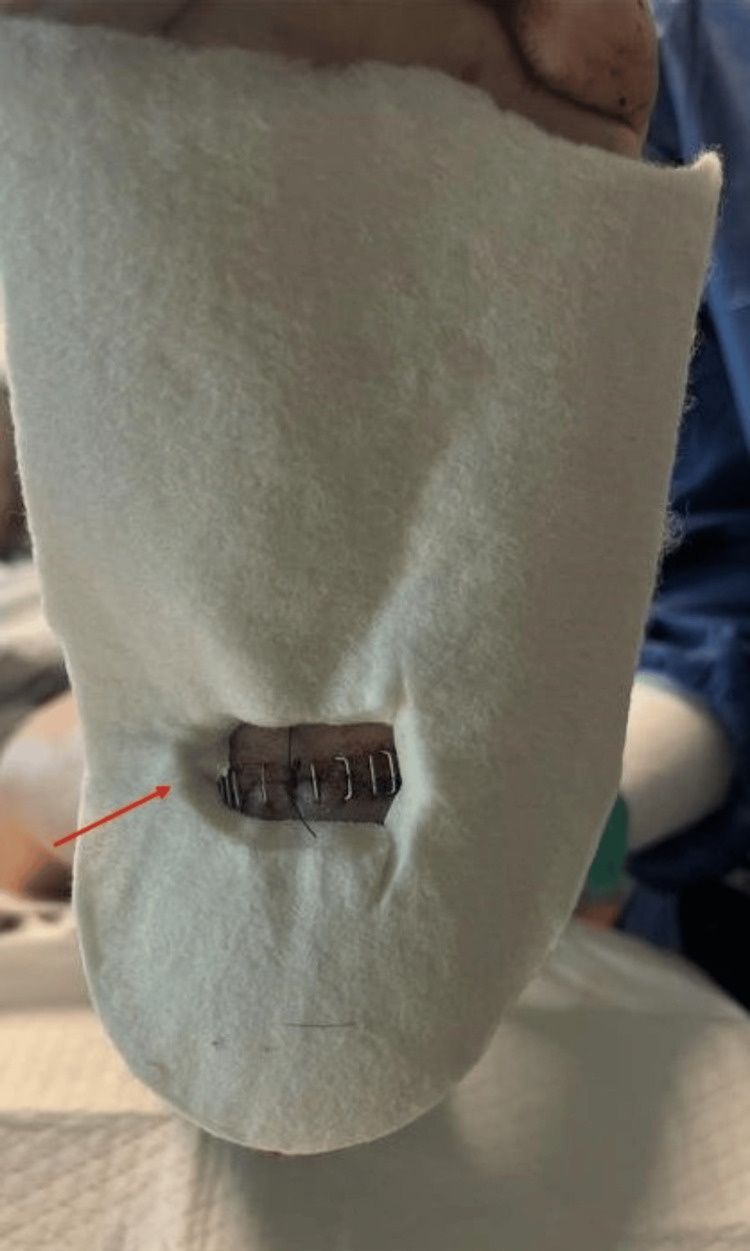
Custom offloading pad for postoperative protection A custom offloading felt pad is applied to the plantar aspect of the foot to offload pressure and protect the incision site. The red arrow indicates a deliberately created aperture in the pad to offload the area directly overlying the surgical sites.

Postoperatively, the patient remained non-weight-bearing in a controlled ankle motion (CAM) walker, followed by gradual progression to partial and full weight-bearing by three weeks. During the initial rehabilitation period, custom offloading felt pads, as seen in Figure 8, were applied to the plantar aspect of both feet to reduce pressure on the surgical site during weight-bearing.

Physical therapy was initiated for the patient after suture removal, focusing on transfers, core strengthening, balance, and assistive device training.

Pain management was an essential component of postoperative recovery. A multimodal pain regimen was prescribed, consisting of scheduled acetaminophen, short-term opioid therapy, baclofen, and gabapentin for neurogenic pain symptoms. The patient was encouraged to elevate and use cold therapy intermittently to minimize swelling.

Functional progress included safe transfer training within the first two weeks, followed by initiation of ambulation with a walker. By three weeks postoperatively, the patient began weight-bearing for transfers and gradually progressed to assisted ambulation. By nine months postoperatively, with the support of physical therapy, the patient ambulated approximately 500 feet using a gait belt and walker, necessitated by Parkinson’s-related gait instability and ongoing core weakness. At the 12-month postoperative period, the patient is now participating in aqua therapy and able to ambulate with ankle-foot orthosis (AFO) braces about 700 feet.

The patient was evaluated by a prosthetist, who recommended a custom bilateral double-upright metal AFO affixed to the shoes. This design offers enhanced structural stability and rotational control, which will be beneficial in managing residual internal rotation and weakness. By attaching the brace to the shoe, this device will minimize the stress at the fusion site while improving gait alignment and efficiency.

Gait Analysis

At nine months postoperatively, the patient demonstrated an altered gait pattern consistent with expectations following bilateral pantalar arthrodesis. Gait evaluation showed reduced step and stride length, contributing to slower walking speed and cadence. The patient walked with a narrow base using a walker and an abdominal safety strap. Due to the absence of ankle motion from fusion, he exhibited increased hip and knee flexion during the swing phase to achieve foot clearance. There was reduced heel strike and increased forefoot loading during stance. Additionally, there was internal rotation of the right lower leg at the knee, adducted forefoot positioning, early heel rise, and hip hiking, resulting in a circumductory gait pattern. By the 12-month period, he is using bilateral double-upright metal AFO with significant improvement in gait patterns. There is mild residual internal rotation with circumduction attributed to weak core and hip flexor muscles.

**Figure 9:**
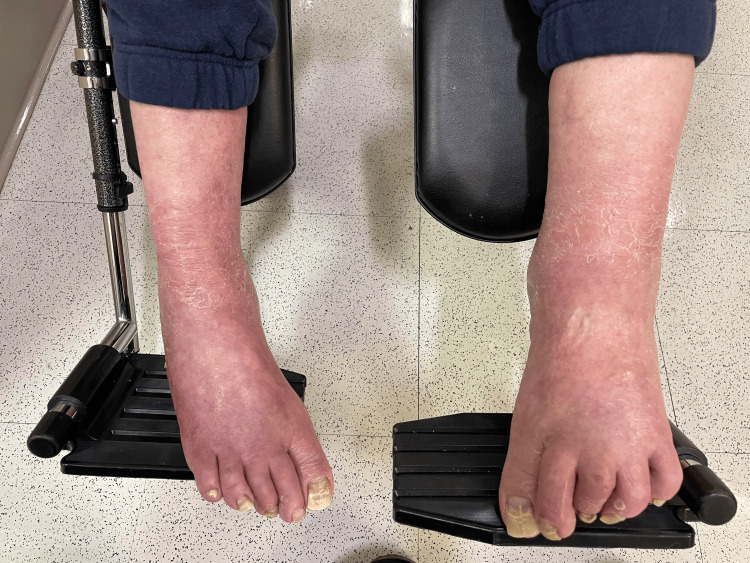
Nine-month postoperative clinical photographs following bilateral pantalar arthrodesis Non-weight-bearing clinical images demonstrate corrected deformity with mild residual forefoot adduction of the right foot. Incision sites are well-healed with no signs of infection, wound dehiscence, or hardware-related complications.

Clinically, bilateral feet were well aligned in a plantigrade position, and incisions were fully healed without signs of infection or skin breakdown. The patient was able to ambulate short distances with a walker, reflecting significant functional improvement from his preoperative non-ambulatory status. Stability during stance and transfers had markedly improved, aligning with his rehabilitation goals. There was mild forefoot abduction noted, but it did not compromise function (Figure 9).

Imaging Outcomes

**Figure 10:**
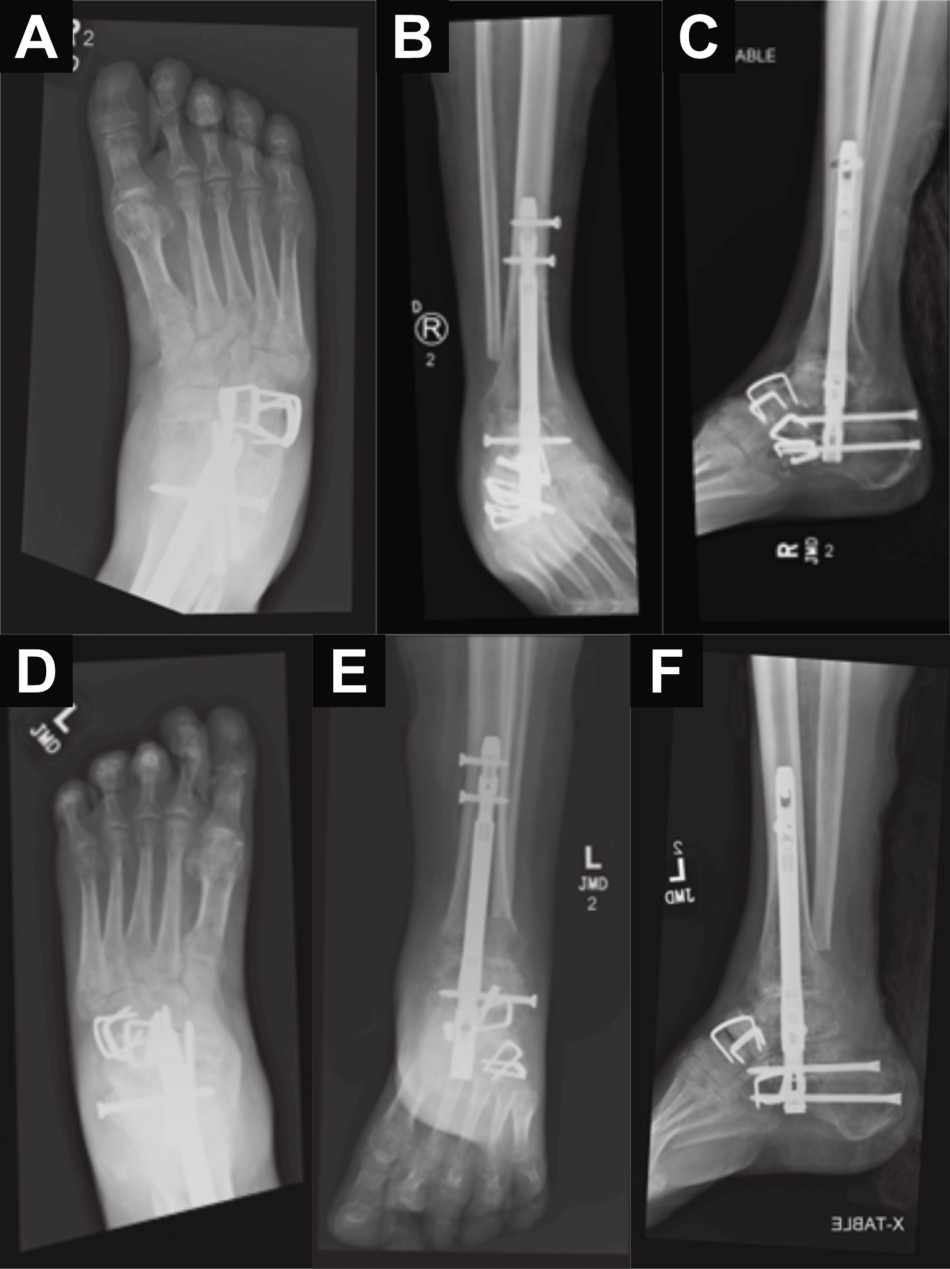
Nine-month postoperative radiographic views following bilateral pantalar arthrodesis A: Right foot anteroposterior view, B: right ankle anteroposterior view, C: right ankle lateral view, D: left foot anteroposterior view, E: left ankle anteroposterior view, F: left ankle lateral view

**Figure 11:**
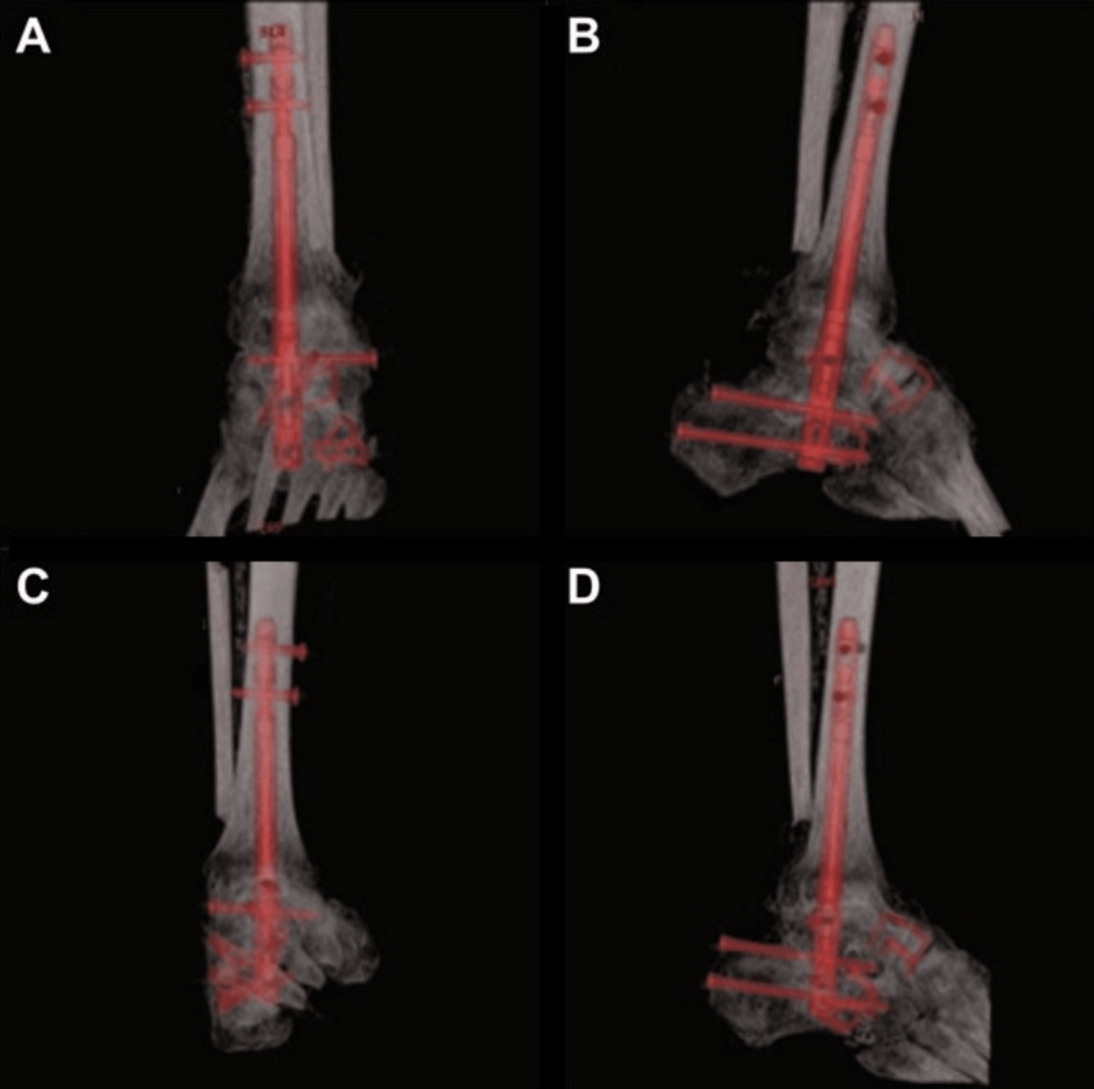
Nine-month postoperative CT scans of bilateral feet A: Coronal view of the left ankle, B: sagittal view of the left ankle, C: coronal view of the right ankle, D: sagittal view of the right ankle The intramedullary nail appears in red on all views to enhance visualization. Osseous fusion is noted across the tibiotalar and subtalar joints bilaterally with maintained alignment and no evidence of hardware complications. CT: computed tomography

Serial radiographs and CT scans were obtained at nine months postoperative, demonstrating successful progression toward fusion and proper alignment of the bilateral hindfoot constructs. Radiographic evaluation (Figure 10A-10F) confirmed placement of intramedullary fixation hardware with no signs of loosening or malposition. Progressive osseous bridging at tibiotalar, talocalcaneal, talonavicular, subtalar, and calcaneocuboid joints were observed. Complementary CT imaging (Figure 11A-11D) provided detailed visualization of all fusion sites, further confirming continuous osseous bridging at the joints. There was no evidence of lucency at the bone implant, nonunion, or hardware failure. These findings reflect successful structural incorporation of the fusion construct and support favorable radiographic outcomes following bilateral pantalar arthrodesis.

Patient-Reported Outcomes

At 12 months postoperatively, the patient reported a notable improvement in pain and function compared to the preoperative baseline. Retrospectively, the patient recalled preoperative pain as severe and sharp, and rated it at 6/10 on the Visual Analog Scale (VAS), leaving the patient wheelchair bound. Postoperatively, pain decreased to 3/10, representing a 50% reduction. Patient satisfaction also improved from 0/10 preoperative to 4/10 postoperatively (Table 2). Functionally, the patient progressed from wheelchair dependence to ambulating approximately 700 feet with a walker and bilateral AFO braces. Overall, he rated the surgical outcome as “better than expected” and expressed moderate satisfaction.

**Table 2:** Postoperative outcomes compared to preoperative baseline VAS and AOFAS Hindfoot scores evaluate pain, function, and alignment preoperatively at six, nine, and 12 months postoperatively. VAS: Visual Analog Scale, AOFAS: American Orthopaedic Foot and Ankle Society

Outcome	Time	Score	Absolute change	% Improvement
VAS pain (0-10)	Preop	6	-	-
	9 months	3	-3	50% reduction
	12 months	3	0	50% reduction
AOFAS Hindfoot (0-100)	Preop	0	-	-
	6 months	39	+39	39% of max
	9 months	49	+49	49% of max
	12 months	49	0	40% of max
Pain subscore (0-40)	Preop	0	-	-
	6 months	20	+20	50% of max
	9 months	30	+30	75% of max
	12 months	30	-	75% of max
Function subscore (0-50)	Preop	0	-	-
	6 months	14	+14	28% of max
	9 months	14	+14	28% of max
	12 months	14	0	28% of max
Alignment subscore (0-10)	Preop	0	-	-
	6 months	5	+5	50% of max
	9 months	5	+5	50% of max
	12 months	5	0	50% of max

The American Orthopaedic Foot and Ankle Society (AOFAS) Hindfoot Scale further reflected these improvements. The preoperative score was 0/100 (pain: 0/40, function: 0/50, alignment: 0/10). At six months, the total improved to 39/100 (pain: 20/40, function: 14/50, alignment: 5/10). By nine months, the total reached 49/100, with pain improving further to 30/40, while function and alignment remained the same. There was no change in the AOFAS score at the 12-month mark. To our knowledge, no minimal clinically important difference (MCID) values have been specifically reported for either the AOFAS Hindfoot Scale or the Visual Analog Scale (VAS) in patients undergoing pantalar arthrodesis. However, a recent study by Paget et al. reported a minimally important change (MIC) of 6.5 points for the patient-reported AOFAS in patients with ankle osteoarthritis [12]. Paget et al. reported a modest improvement from relatively preserved baseline scores (AOFAS: 59.4 to 63.6, pain: 19.7 to 18.1, function: 31.2 to 36.3, alignment: 8.1 to 9.2) [12], whereas our case started from extremely low baseline scores and experienced large gains due to mobility issues (AOFAS: 0 to 49, pain: 0 to 30. function: 0 to 14, alignment: 0 to 5). The large improvement in our patient was mainly reflected in preoperative disability and pain, so direct comparison should be interpreted cautiously. The comparison between these two cases is limited by differing cohorts, indication, study design, and pathology.

Postoperative Complications

The primary postoperative complication involved delayed wound healing over the right posterior ankle, attributed to significant soft tissue tension from preexisting equinovarus contracture. Wound care involved the application of silver sulfadiazine cream and protective padding. No signs of infection or systemic involvement were noted. The wound healed within a month postoperatively.

## Discussion

Literature review

Historical Approach

Pantalar arthrodesis has gained popularity as a limb salvage procedure for severe hindfoot deformities. The primary goal for the procedure is to achieve a plantigrade and functional foot, thereby improving mobility and quality of life. Pantalar arthrodesis was first described by Lorthior in 1911 as a treatment for severe equinovarus deformity. His technique involved removal of the talus, denudation of the cartilaginous surfaces, and reinsertion of the talus as a free bone graft to achieve fusion across the hindfoot and ankle joints [5]. As surgical techniques advanced, it became clear that the tibiotalar and subtalar joints provided the backbone of stability in pantalar fusion, which led to the development of the more targeted tibiotalocalcaneal (TTC) arthrodesis. The paper by Woods et al. summarized the evolution of IM nail fixation, highlighting key developments in the techniques over time. The first description of IM fixation for ankle arthrodesis was provided by Lexer in 1906, whose team used boiled corpse bone as a graft, which was placed through the calcaneus, talus, and tibia [13]. In 1948, Adams published the first clinical results of ankle arthrodesis using a retrograde metallic nail [14]. This offered a more reliable method of joint fixation. By the early 1990s, IM nails with interlocking screws became popular for hindfoot arthrodesis to provide additional stability [15]. Given the historical progression of IM nail fixation and its biomechanical advantages, this technique was chosen in our patient to address bilateral equinovarus deformity in the setting of Parkinson’s disease and osteopenia.

Single Stage Versus Staged

While deciding between single-stage or staged bilateral pantalar arthrodesis, unilateral procedures are more common and studied, but bilateral cases remain less reported, complicating clinical decision-making. A staged approach, where each limb is operated on separately, offers the advantage of more gradual recovery and allows the contralateral limb to support rehabilitation. However, this method may prolong the recovery period and expose patients to multiple episodes of anesthesia and extended hospitalization. Prior studies advocate for staging of pantalar arthrodesis due to increased risk of avascular necrosis, malunion, nonunion, and wound problems [16]. However, in our case, we did not experience those complications. Additionally, patients with bilateral deformity may experience compensation-related complications or overuse injuries between procedures. In contrast, single-stage bilateral pantalar arthrodesis addresses both deformities, offering a unified recovery trajectory, reducing overall surgical burden, and decreasing the risk of deep vein thrombosis and pressure ulcers. This approach is particularly beneficial for patients with progressive neuromuscular conditions, such as Parkinson’s disease, where delaying correction may lead to irreversible contractures or functional decline. In this case, the patient’s declining mobility, history of Parkinson’s disease, and strong caregiver support favored a single-stage procedure. The patient and family were counseled extensively on the risks, benefits, and expected postoperative course, and elected for a single hospitalization and consolidated rehabilitation period. Ultimately, thorough medical history, patient selection, and setting clear expectations are key to successful single-stage bilateral pantalar arthrodesis.

Biomechanics of IM Nail Fixation in TTC (Within Pantalar Arthrodesis)

There are multiple methods of fixation that have been described for TTC arthrodesis, such as staples, screws, plates, and IM nails. Among these, the IM nail technique has demonstrated a high fusion rate for complex ankle arthrodesis cases, including 86% at 14 weeks [17] and 84% at 16 weeks [18]. With the current literature, this suggests that TTC arthrodesis with IM nail is an effective surgical option for achieving joint fusion within 3.5 months. Although the IM nail specifically stabilizes the tibiotalar and subtalar joints, pantalar arthrodesis additionally requires fusion of the talonavicular and calcaneocuboid joints, which must be addressed through supplemental fixation.

Biomechanical studies further support the advantage of IM nail fixation over other methods. Berend et al. found that IM nail fixation was significantly stiffer than the cross lag-screw construct after undergoing bending and torsional tests. The IM nail provided superior biomechanical stability across all tested directions, including plantarflexion, dorsiflexion, inversion, eversion, and rotational forces [8]. These findings suggest that increased stiffness can contribute to improved fusion rates and better maintenance of hindfoot alignment during the healing process. Noonan et al. studied the biomechanical effects of nail length in TTC arthrodesis. In this cadaveric study, they found that longer IM nails reduce proximal tibial stress and mitigate the risk of fatigue fracture, particularly in patients with osteopenia [19]. Moore et al. further emphasized that in severe osteopenic bone, the use of IM nail for ankle arthrodesis allows for early weight-bearing due to its stable fixation modality [20]. Early weight-bearing is crucial as it helps prevent disuse osteopenia, reduces the risk of stress fractures, promotes muscle strength, and enhances bone healing through mechanical loading as described by Wolff’s law. Furthermore, a retrospective study of 42 ankles demonstrates a 97.6% union rate overall, but plate fixation was associated with higher infection rates compared to screw or intramedullary nail fixation, where no infections were observed. Furthermore, intramedullary nail fixation was reported to have the lowest complication rates, with prior biomechanical studies confirming superior construct stiffness compared to cross screws [21]. In our case, the IM nail was provided with the primary biomechanical stability for the TTC component, while the talonavicular and calcaneocuboid joints were fused for additional correction and to achieve pantalar arthrodesis. The patient had been non-ambulatory for several years due to advanced Parkinson’s disease and a lumbar compression fracture, which progressively impacted mobility. The decision to proceed with a single-stage bilateral pantalar arthrodesis using IM nail was made with careful consideration of the patients’ complex clinical history and presentation. The IM nail’s superior biomechanical stability was crucial to accommodate the patient’s compromised bone quality and reduced risk of muscle atrophy and complications such as DVT and pressure ulcers. The IM nail stability accommodated the patient’s poor bone quality and facilitated early rehabilitation to prevent postoperative complications.

Postoperative Management

Postoperative management encompasses rehabilitation, pain control, and monitoring potential complications. Early weight-bearing and mobility are important for patients undergoing pantalar arthrodesis to prevent muscle atrophy, compensatory gait, deep vein thrombosis (DVT), and pressure ulcers. From a functional standpoint, it supports a faster recovery, prevents deconditioning, and facilitates progression to a better quality of life. Due to the patient’s underlying Parkinson’s disease, initiation of physical therapy was closely coordinated to optimize therapy and minimize complications. Three months postoperatively, the patient utilized a walker and belt strap for additional support and added safety. Physical therapy primarily focused on gait training, balance, and core strengthening to allow independent transfers and ambulation. 

Effective rehabilitation following bilateral pantalar arthrodesis relies not only on physical recovery but also on pain control to ensure patient comfort during therapy. A multimodal pain regimen was used for a safe and comfortable rehabilitation period. Vesely et al. analyzed the impact of pain after TTC arthrodesis and found that patients with a diagnosis of chronic pain have difficulty with postoperative pain control and may require a multidisciplinary approach [22]. This is an important discussion to have preoperatively to set realistic pain management expectations. Additionally, their study found that older patients experienced greater postoperative pain improvement, suggesting a negative correlation between age and pain levels. However, in this case report, the patient underwent bilateral pantalar arthrodesis and received extensive multimodal pain management. Furthermore, Parkinson’s disease can contribute to pain through mechanisms such as muscle rigidity, dystonia, and altered pain processing pathways, which may complicate postoperative recovery. Given the patient’s underlying Parkinson’s disease and neurological impairment, functional recovery was expectedly limited, which is reflected in the lower functional AOFAS scores despite meaningful improvement in pain.

While pantalar arthrodesis is an effective surgical option to manage complex hindfoot deformities, it carries risks of complications. These can range from surgical site infection or wound dehiscence, nonunion, and malunion to hardware failure or breakage [6,18]. In a multicenter study of TTC arthrodesis, only 24% experienced complications during the postoperative period to final follow-up. Four patients required a revisional surgery for nonunion, in which the implant was removed and bone grafts were incorporated to promote healing and fusion [18]. In this case report, this patient experienced a delay in wound healing, which was attributed to the severe fixed equinus contracture present significantly in the right ankle compared to the left ankle. To address the contracture, a decision was made to perform a Z-plasty to allow adequate skin re-approximation. Additionally, a Neox RT amniotic allograft was applied to the site of the Z-plasty to support skin healing. Despite this preoperative planning, the patient still experienced some delay in wound healing on the posterior aspect of the right ankle. This complication was managed with local wound care and offloading.

Clinical Implication

This case highlights the utilization of retrograde IM nail fixation to manage complex bilateral deformities. A thorough patient history and patient selection are crucial when opting for bilateral pantalar arthrodesis. Factors such as comorbidities, bone quality, support system, and goals must be evaluated and discussed to ensure expectations are aligned and met. The strengths of this case include the detailed surgical technique, successful outcomes despite multiple comorbidities, and the novelty of this clinical presentation. While VAS and AOFAS scores were reported, no validated patient-reported outcome measures (PROMs) assessing quality of life (QOL) or activities of daily living (ADL) were used, which limits the evaluation of postoperative recovery from the patient’s perspective. Additionally, the follow-up period in this case is limited to nine months; although the short-term results are promising, the lack of long-term follow-up introduces uncertainty regarding the durability of fusion and sustained functional outcomes. The need for long-term studies and more outcome measures on bilateral pantalar outcomes is necessary to refine technique, optimize patient selection, and enhance postoperative protocols.

## Conclusions

This case reinforces the importance of a thorough patient evaluation, careful surgical planning, and a multidisciplinary approach to the management of complex bilateral hindfoot deformity. Single-stage bilateral pantalar arthrodesis served as a limb salvage option that achieved pain relief, alignment correction, and improved mobility in the short term. However, given the single-patient nature of this report, limited follow-up time, and modest functional recovery, findings should be interpreted as preliminary. Further studies with a larger cohort and longer-term outcomes are needed to determine the generalizability and durability of this approach.

## References

[1] Varghese G, Redford JB (2000). Nerve and muscle disorders and their sequelae. Foot Ankle Clin.

[2] Wong CC, McGirt MJ (2013). Vertebral compression fractures: a review of current management and multimodal therapy. J Multidiscip Healthc.

[3] Tedeschi R (2023). Automated mechanical peripheral stimulation for gait rehabilitation in Parkinson's disease: a comprehensive review. Clin Park Relat Disord.

[4] Martin KD, Jastifer J, Scott D, Grzeskiewicz E (2024). Spastic equinovarus foot deformity. J Am Acad Orthop Surg.

[5] Mandracchia VJ, Mandi DM, Nickles WA, Barp EA, Sanders SM (2004). Pantalar arthrodesis. Clin Podiatr Med Surg.

[6] Thomas RL, Sathe V, Habib SI (2012). The use of intramedullary nails in tibiotalocalcaneal arthrodesis. J Am Acad Orthop Surg.

[7] Hsu AR, Ellington JK, Adams SB Jr (2015). Tibiotalocalcaneal arthrodesis using a nitinol intramedullary hindfoot nail. Foot Ankle Spec.

[8] Berend ME, Glisson RR, Nunley JA (1997). A biomechanical comparison of intramedullary nail and crossed lag screw fixation for tibiotalocalcaneal arthrodesis. Foot Ankle Int.

[9] Bennett GL, Cameron B, Njus G, Saunders M, Kay DB (2005). Tibiotalocalcaneal arthrodesis: a biomechanical assessment of stability. Foot Ankle Int.

[10] Belczyk R, Sung W, Wukich Dane K (2008). Technical tip: a simple method for proper placement of an intramedullary nail entry point for tibiotalocalcaneal or tibiocalcaneal arthrodesis. FAOJ.

[11] Kitaoka HB, Alexander IJ, Adelaar RS, Nunley JA, Myerson MS, Sanders M (1994). Clinical rating systems for the ankle-hindfoot, midfoot, hallux, and lesser toes. Foot Ankle Int.

[12] Paget LD, Sierevelt IN, Tol JL, Kerkhoffs GM, Reurink G (2023). The completely patient-reported version of the American Orthopaedic Foot and Ankle Society (AOFAS) score: a valid and reliable measurement for ankle osteoarthritis. J ISAKOS.

[13] Lexer E (2008). The use of free osteoplasty together with trials on arthrodesis and joint transplantation. Archiv für klin Chirurgie. 1908;86(4):939-954. Clin Orthop Relat Res.

[14] Adams Adams, JC JC (1948). Arthrodesis of the ankle joint; experiences with the transfibular approach. J Bone Joint Surg Br.

[15] Woods JB, Burns PR (2011). Advances in intramedullary nail fixation in foot and ankle surgery. Clin Podiatr Med Surg.

[16] Acosta R, Ushiba J, Cracchiolo A 3rd (2000). The results of a primary and staged pantalar arthrodesis and tibiotalocalcaneal arthrodesis in adult patients. Foot Ankle Int.

[17] Chou LB, Mann RA, Yaszay B (2000). Tibiotalocalcaneal arthrodesis. Foot Ankle Int.

[18] Rammelt S, Pyrc J, Agren PH (2013). Tibiotalocalcaneal fusion using the hindfoot arthrodesis nail: a multicenter study. Foot Ankle Int.

[19] Noonan T, Pinzur M, Paxinos O, Havey R, Patwardhin A (2005). Tibiotalocalcaneal arthrodesis with a retrograde intramedullary nail: a biomechanical analysis of the effect of nail length. Foot Ankle Int.

[20] Moore TJ, Prince R, Pochatko D, Smith JW, Fleming S (1995). Retrograde intramedullary nailing for ankle arthrodesis. Foot Ankle Int.

[21] van den Heuvel SB, Penning D, Schepers T (2022). Open ankle arthrodesis: a retrospective analysis comparing different fixation methods. J Foot Ankle Surg.

[22] Vesely BD, Kipp J, Russell G, LeSavage L, Hoffler H, Medda AW, Scott AT (2024). Predictive factors of postoperative pain in patients with tibiotalocalcaneal arthrodesis with an intramedullary nail: a retrospective review. J Foot Ankle Surg.

